# Active inference, sensory attenuation and illusions

**DOI:** 10.1007/s10339-013-0571-3

**Published:** 2013-06-07

**Authors:** Harriet Brown, Rick A. Adams, Isabel Parees, Mark Edwards, Karl Friston

**Affiliations:** 1 Institute of Neurology, The Wellcome Trust Centre for Neuroimaging, UCL, 12 Queen Square, London, WC1N 3BG UK; 2 The Sobell Department of Motor Neuroscience and Movement Disorders, UCL, 33 Queen Square, London, WC1N 3BG UK

**Keywords:** Free energy, Active inference, Sensory attenuation, Illusion, Attention, Schizophrenia

## Abstract

Active inference provides a simple and neurobiologically plausible account of how action and perception are coupled in producing (Bayes) optimal behaviour. This can be seen most easily as minimising prediction error: we can either change our predictions to explain sensory input through *perception*. Alternatively, we can actively change sensory input to fulfil our predictions. In active inference, this *action* is mediated by classical reflex arcs that minimise proprioceptive prediction error created by descending proprioceptive predictions. However, this creates a conflict between action and perception; in that, self-generated movements require predictions to override the sensory evidence that one is not actually moving. However, ignoring sensory evidence means that externally generated sensations will not be perceived. Conversely, attending to (proprioceptive and somatosensory) sensations enables the detection of externally generated events but precludes generation of actions. This conflict can be resolved by attenuating the precision of sensory evidence during movement or, equivalently, attending away from the consequences of self-made acts. We propose that this Bayes optimal withdrawal of precise sensory evidence during movement is the cause of psychophysical sensory attenuation. Furthermore, it explains the force-matching illusion and reproduces empirical results almost exactly. Finally, if attenuation is removed, the force-matching illusion disappears and false (delusional) inferences about agency emerge. This is important, given the negative correlation between sensory attenuation and delusional beliefs in normal subjects—and the reduction in the magnitude of the illusion in schizophrenia. Active inference therefore links the neuromodulatory optimisation of precision to sensory attenuation and illusory phenomena during the attribution of agency in normal subjects. It also provides a functional account of deficits in syndromes characterised by false inference and impaired movement—like schizophrenia and Parkinsonism—syndromes that implicate abnormal modulatory neurotransmission.

## Highlights

- Sensory attenuation is necessary for behaviour under active (Bayesian) inference.
- Sensory attenuation can be understood as the attenuation of sensory precision.
- A failure of sensory attenuation leads to false inference and beliefs about agency.
- This provides a normative account of the force-matching illusion in schizophrenia.

## Introduction

Children discover early in life that although they can tickle others and be tickled by others, it is almost impossible to tickle oneself. The commonplace nature of this observation hides its profundity—two physically identical sensory stimuli can be perceived differently, depending on high-level concepts such as agency or wilfulness. This sort of effect has now been quantified in a number of tasks and has been investigated in numerous neuroimaging studies. However, after more than a decade of research, a simple explanation is still outstanding. In this paper, we try to provide a principled account of how beliefs about agency depend upon the active sampling of sensory information (active inference), and how this leads naturally to phenomena like sensory attenuation, the force-matching illusion and attribution of agency.

### Sensory attenuation and agency

The difference between self-generated and externally generated tickle has been the focus of many studies (Weiskrantz et al. 1971; Claxton 1975; Blakemore et al. 1999). Self-produced tickle is consistently rated less ‘ticklish’ than externally produced tickle, and its ticklishness can be increased by closing the eyes (Claxton 1975). Tickle is not the only attribute of sensation affected—self-generated touch stimuli are also perceived as less pleasant and intense (Blakemore et al. 1999). Indeed, sensory attenuation is not limited to somatosensation; attenuation of self-generated visual (Hughes and Waszak 2011; Cardoso-Leite et al. 2010) and auditory sensations have been reported (Martikainen et al. 2005; Weiss et al. 2011a, b; Desantis et al. 2012).

A measure of sensory attenuation is provided by the force-matching task (Shergill et al. 2003, 2005). During this task, instead of reporting sensations explicitly, subjects match a reference force, either by pressing directly on themselves, or by using a robot to reproduce the perceived pressure. Higher levels of matched force are produced when the force is self-generated, consistent with self-reports of sensory attenuation.

Sensory attenuation is also evident in neuronal responses. Subcortically, both cerebellar (Blakemore et al. 1998, 1999a, b, 2001) and thalamic (Blakemore et al. 1998) activity is reduced for self-produced versus externally produced sensations. Early sensory responses are also modulated in auditory paradigms, where these differences can appear as early as 27 ms after stimulus onset (Baess et al. 2008, 2009; Aliu et al. 2009; Martikainen et al. 2005). Repetitive stimulation of M1 (which has a depressive effect on activity) reduces the magnitude of sensory attenuation in the force-matching task, as well as in a grip-production task (Therrien et al. 2011; Voss et al. 2007), whereas single-pulse TMS of M1 just before movement onset (which delays the movement) has no effect on the level of sensory attenuation (Voss et al. 2006). This suggests that M1 is involved in determining the level of sensory attenuation but not in mediating it. In visual studies, the only ERP change noted thus far is a late (~150 ms) modulation of frontoparietal potentials (Schafer and Marcus 1973; Hughes and Waszak 2011). Concepts, such as meaning, perception of agency and social factors, can influence sensory attenuation. Curio et al. (2000) demonstrated the absence of the late (300 ms) ‘oddball’ potentials (usually elicited in response to rare stimuli which have ‘meaning’ or task relevance) in response to self-generated stimuli, suggesting that they are categorised as distinct from externally generated stimuli at a conceptual level. Sato (2008) observed sensory attenuation both when participants performed a movement resulting in a tone, and when they observed experimenters performing the same movement. Similarly, Weiss et al. (2011a, b) noted greater sensory attenuation when participants triggered the experimenter to produce externally generated tones by tapping them and vice versa for self-generated tones.

The relationship between sensory attenuation and the experience of agency is complex. An experience of agency over movements that generate sensation seems to be necessary for sensory attenuation (Desantis et al. 2012; Gentsch and Schütz-Bosbach 2011): sensory attenuation does not occur if movement and sensation are correlated, but the relationship is not perceived as causal. Some authors have suggested that the experience of sensory attenuation is important in labelling movements as self-generated (Blakemore et al. 2002). In support of this idea, Baess et al. (2011) found that sensory attenuation was more pronounced in blocks with mixed self- and externally produced sensations. In this setting, the attribution of agency is more difficult than during a sequence of sensations that are purely self- or purely externally generated.

Sensory attenuation is an interesting phenomenon partly because sensory attenuation is reduced in schizophrenia (Blakemore et al. 2000; Shergill et al. 2005), or those at high risk of developing psychosis (Wilquin and Delevoye-Turrell 2012). In normal subjects, sensory attenuation is (negatively) correlated with the level of delusional beliefs (Teufel et al. 2010). Less sensory attenuation means that the percepts of schizophrenics are more veridical than controls and—in the force-matching task—they perform more accurately (Shergill et al. 2005). This means that differences between schizophrenics and controls are difficult to attribute to non-specific effects of long-term disease, psychoactive medication or social deprivation, and that investigating this effect might provide clues about the pathogenesis of schizophrenia. A key symptom of schizophrenia is aberrant perception of agency (Frith 2005), particularly the delusion that one’s actions are being controlled by others, suggesting the mechanisms that impair sensory attenuation in schizophrenia are intimately related to the perception of agency.

### Formal theories of sensory attenuation

Previous explanations for the force-matching paradigm—that can be applied to sensory attenuation more generally—have come from engineering approaches to motor control (Wolpert and Flanagan 2001). In the model proposed by Blakemore et al. (1999b), the decision to move initiates a motor command, which is transformed by a forward model into a prediction of the sensations created by that movement. The real sensations produced by the movement are compared to the predictions of the forward model to produce a ‘control theory’ prediction error, which is used to update predictions and refine the forward model. During self-generated movement, an accurate forward model means that there is little prediction error. Under this model, it is suggested that small prediction errors during self-generated movement lead to a percept of a less intense force, relative to the true force.

This model is incomplete in a number of aspects. Firstly, it is unclear why the intensity of a percept is related to the size of prediction error: prediction errors are used to update predictions, but they do not constitute predictions or percepts per se. Within predictive coding formulations of perception (Rao and Ballard 1999; Friston 2005), prediction errors play a crucial role in perception, but again, they are not the percept itself; the percept is a synthesis of prior beliefs and sensory evidence that is conveyed by prediction errors.

Second, this explanation overlooks the multidimensional nature of sensory attributes. In the optimal control explanation, any mismatch between the forward model and sensory input is mapped to a single variable that determines perceived intensity. It is true that parametrically varying the time delay between movement and sensation—or rotating sensory feedback with respect to movement—will alter the force-matching illusion (Blakemore et al. 1999a, b). However, the optimal control formulation does not explain how this is caused by the amplitude of prediction error, pooled over all sensory channels. Furthermore, the amplitude of prediction error does not seem to be important in determining the level of sensory attenuation: for example, Baess et al. (2008) show that the predictability of a self-generated sensation does not affect sensory attenuation. Crucially, a self-generated movement that should result in sensation—but does not—can still cause sensory attenuation, despite the implicit production of prediction errors (Bays et al. 2005).

Third, there is a set of results that control theory approach cannot account for. During self-generated movement, sensory attenuation is often noted in response to *externally* generated stimuli (Voss et al. 2008; Rushton et al. 1981; Milne et al. 1988; Chapman et al. 1987). These stimuli are applied by the experimenter, so they cannot be predicted by the forward model and therefore cannot be attenuated. Additionally, sensory attenuation has been found for stimuli that occur (up to 400 ms) before the onset of movement (Voss et al. 2008; Bays et al. 2005) when they cannot be predicted from self-generated movement. This attenuation seems to be due to changes in sensitivity (*d*-prime) to external stimuli rather than a change in the response criterion (Juravle and Spence 2011; Van Hulle et al. 2013). The attenuation of these stimuli—which cannot be predicted from motor commands—suggests that the phenomenon of sensory attenuation is broader than suggested by optimal control formulations.

In this paper, we propose an alternative explanation for sensory attenuation based on active inference. Active inference is based on Bayes optimal accounts of behaviour and provides a principled explanation of how sensory attenuation may arise in a Bayes optimal (normative) sense. This is in contrast to previous explanations, which have explained sensory attenuation as a quirk or anomaly of motor control. Instead, we suggest that sensory attenuation is a necessary consequence of reducing the precision of sensory evidence during movement to allow the expression of proprioceptive predictions that incite movement. This explanation is potentially important because a failure of sensory attenuation may result in false inference about the causes (agency) of self-made acts—a failure that is characteristic of the positive symptoms of schizophrenia. Furthermore, the neuronal mechanisms behind sensory attenuation (and compensatory changes in the precision of beliefs at non-sensory levels) rest on neuromodulatory mechanisms that have been implicated in psychosis.

In the following, we summarise active inference and its neurobiological implementation. This implementation is used in later simulations to demonstrate why sensory attenuation is necessary for movement. We then simulate the force-matching illusion using exactly the same scheme. We conclude by simulating a loss of sensory attenuation and a compensatory increase in non-sensory precision, as might be found in schizophrenia. Crucially, this simulated pathology exposes actors to false beliefs or delusions, interestingly, with a necessarily antagonistic content. These simulations do not model all the aspects of sensory attenuation discussed above (e.g. Sato 2008); however, we hope that the principles of active inference—in particular, the optimisation of precision at different levels of a predictive coding hierarchy—may generalise to other settings.

## Neurobiological implementation of active inference

We start by considering how active inference might be implemented in the brain. The results of this normative treatment are differential equations that describe neuronal activity and ensuing action, which we then use to demonstrate the necessary role of sensory attenuation and the illusory phenomena that it entails. The equations may appear a bit complicated, but they are based on just three assumptions:

- The brain minimises the free energy of sensory inputs defined by a generative model.
- The generative model used by the brain is hierarchical, nonlinear and dynamic.
- Neuronal firing rates encode the expected state of the world, under this model.

The first assumption is the free energy principle, which leads to active inference in the embodied context of action. This provides a principled (Bayes optimal) explanation for action and perception, in which both minimise a free energy bound on the (negative) Bayesian log evidence for a generative model of the sensorium. This means that minimising free energy maximises Bayesian model evidence. The second assumption—about the nature of the models entailed by neuronal circuits—is motivated easily by noting that the world is both dynamic and nonlinear and that hierarchical causal structure emerges inevitably from a separation of temporal scales (Ginzburg and Landau 1950; Haken 1983). The final assumption is the Laplace assumption that, in terms of neural codes, leads to the Laplace code that is arguably the simplest and most flexible of all neural codes (Friston 2009).

Given these assumptions, one can simulate a whole variety of neuronal processes by specifying the particular equations that constitute a generative model. The resulting perception and action are specified completely by the above assumptions and can be implemented in a biologically plausible way as described below. In brief, these simulations use differential equations that minimise the free energy of sensory input using a generalised gradient descent (Friston et al. 2010a, b).

1 $$ \begin{gathered} \dot{\tilde{\mu }}(t) = \mathcal{D}\tilde{\mu }(t) - \partial_{{\tilde{\mu }}} F(\tilde{s},\tilde{\mu }) \\ \dot{a}(t) = - \partial_{a} F(\tilde{s},\tilde{\mu }) \\ \end{gathered} $$

These coupled differential equations describe perception and action, respectively, and just say that neuronal activity encoding conditional expectations $\tilde{\mu }\ominus = (\mu ,\mu^{\prime},\mu^{\prime\prime}, \ldots )$ and action $a$ change to reduce free energy, where free energy $F(\tilde{s},\tilde{\mu })$ is a function of sensory inputs $\tilde{s} = (s,s\prime ,s\prime \prime , \ldots )$ and conditional expectations encoded by neuronal activity. The first differential equation has the same form as Bayesian (e.g., Kalman-Bucy) filters used in time series analysis. The first term is a prediction based upon a differential matrix operator $\mathcal{D}$ that returns the generalised motion of the expectation. The second (correction) term is usually expressed as a mixture of prediction errors that ensures the changes in conditional expectations are Bayes optimal predictions about hidden states of the world.

The second differential equation says that action also minimises free energy. The differential equations above are coupled because sensory input depends upon action, which depends upon perception through the conditional expectations. This circular dependency leads to a sampling of sensory input that is both predicted and predictable, thereby minimising free energy and prediction errors.

To perform neuronal simulations under this scheme, it is only necessary to integrate or solve Eq. (1) to simulate the neuronal dynamics that encode conditional expectations and the ensuing action. Conditional expectations depend upon the brain’s generative model of the world, which we assume has the following hierarchical form

2 $$ \begin{array}{*{20}c}    {s = g^{{(1)}} (x^{{(1)}} ,v^{{(1)}} ) + \omega _{v}^{{(1)}} }  \\    {\dot{x}^{{(1)}}  = f^{{(1)}} (x^{{(1)}} ,v^{{(1)}} ) + \omega _{x}^{{(1)}} }  \\     \vdots   \\    {v^{{(i - 1)}}  = g^{{(i)}} (x^{{(i)}} ,v^{{(i)}} ) + \omega _{v}^{{(i)}} }  \\    {\dot{x}^{{(i)}}  = f^{{(i)}} (x^{{(i)}} ,v^{{(i)}} ) + \omega _{x}^{{(i)}} }  \\     \vdots   \\    {\omega _{x}^{{(i)}} \sim N(0,\Pi _{x}^{{(i) - 1}} )}  \\    {\omega _{v}^{{(i)}} \sim N(0,\Pi _{v}^{{(i) - 1}} )}  \\    {\Pi _{x}^{{(i)}}  = {\text{diag}}\left( {\exp \left( {\pi _{x}^{{(i)}} \left( {x^{{(i)}} ,v^{{(i)}} } \right)} \right)} \right)}  \\    {\Pi _{v}^{{(i)}}  = {\text{diag}}\left( {\exp \left( {\pi _{v}^{{(i)}} \left( {x^{{(i)}} ,v^{{(i)}} } \right)} \right)} \right)}  \\   \end{array} $$

This equation is just a way of specifying a generative model in terms of a probability density over the sensory and hidden states, where the hidden states have been divided into hidden states and causes $(x^{(i)} ,\,v^{(i)} )$. Here, $(g^{(i)},\,f^{(i)} )$ are nonlinear functions of hidden states that generate sensory inputs at the first level. Random fluctuations $\left( {\omega_{x}^{(i)} ,\omega_{v}^{(i)} } \right)$ in the hidden causes and motion of states enter each level of the hierarchy. Gaussian assumptions about these random fluctuations make the model probabilistic—they play the role of sensory noise at the first level and induce uncertainty at higher levels. The amplitudes of these random fluctuations are quantified by their precisions $(\Uppi_{x}^{(i)} ,\Uppi_{v}^{(i)} )$ that may depend upon the hidden states or causes through log precisions $(\pi_{x}^{(i)} ,\pi_{v}^{(i)} )$. Hidden causes link hierarchical levels, whereas hidden states link dynamics over time. Hidden states and causes are abstract quantities (like the motion of an object in the field of view) that the brain uses to explain or predict sensations.

### Perception and predictive coding

Given the form of the generative model (Eq. 2), we can now write down the differential equations (Eq. 1) describing neuronal dynamics in terms of (precision weighted) prediction errors on the hidden causes and states. These errors represent the difference between conditional expectations and predicted values, under the generative model (using $A \cdot B: = A^{T} B$ and omitting higher order terms):

3 $$ \begin{aligned} \dot{\tilde{\mu }}_{x}^{(i)} & = \mathcal{D}\tilde{\mu }_{x}^{(i)} + \left( {\frac{{\partial \tilde{g}^{(i)} }}{{\partial \tilde{\mu }_{x}^{(i)} }} - \tfrac{1}{2}\tilde{\varepsilon }_{v}^{(i)} \cdot \frac{{\partial \tilde{\Upomega }_{v}^{(i)} }}{{\partial \tilde{\mu }_{x}^{(i)} }}} \right) \cdot \xi_{v}^{(i)} + \left( {\frac{{\partial \tilde{f}^{(i)} }}{{\partial \tilde{\mu }_{x}^{(i)} }} - \tfrac{1}{2}\tilde{\varepsilon }_{x}^{(i)} \cdot \frac{{\partial \tilde{\Upomega }_{x}^{(i)} }}{{\partial \tilde{\mu }_{x}^{(i)} }}} \right) \cdot \xi_{x}^{(i)} + \frac{{\partial tr\left( {\tilde{\Upomega }_{v}^{(i)} + \tilde{\Upomega }_{x}^{(i)} } \right)}}{{\partial \tilde{\mu }_{x}^{(i)} }} - \mathcal{D}^{T} \xi_{x}^{(i)} \\ \dot{\tilde{\mu }}_{v}^{(i)} & = \mathcal{D}\tilde{\mu }_{v}^{(i)} + \left( {\frac{{\partial \tilde{g}^{(i)} }}{{\partial \tilde{\mu }_{v}^{(i)} }} - \tfrac{1}{2}\tilde{\varepsilon }_{v}^{(i)} \cdot \frac{{\partial \tilde{\Upomega }_{v}^{(i)} }}{{\partial \tilde{\mu }_{v}^{(i)} }}} \right) \cdot \xi_{v}^{(i)} + \left( {\frac{{\partial \tilde{f}^{(i)} }}{{\partial \tilde{\mu }_{x}^{(i)} }} - \tfrac{1}{2}\tilde{\varepsilon }_{x}^{(i)} \cdot \frac{{\partial \tilde{\Upomega }_{x}^{(i)} }}{{\partial \tilde{\mu }_{v}^{(i)} }}} \right) \cdot \xi_{x}^{(i)} + \frac{{\partial tr\left( {\tilde{\Upomega }_{v}^{(i)} + \tilde{\Upomega }_{x}^{(i)} } \right)}}{{\partial \tilde{\mu }_{v}^{(i)} }} - \xi_{v}^{(i + 1)} \\ \xi_{x}^{(i)} & = \tilde{\Uppi }_{x}^{(i)} \tilde{\varepsilon }_{x}^{(i)} = \Uppi_{x}^{(i)} \left( {\mathcal{D}\tilde{\mu }_{x}^{(i)} - \tilde{f}^{(i)} \left( {\tilde{\mu }_{x}^{(i)} ,\tilde{\mu }_{v}^{(i)} } \right)} \right) \\ \xi_{v}^{(i)} & = \Uppi_{v}^{(i)} \tilde{\varepsilon }_{v}^{(i)} = \Uppi_{v}^{(i)} \left( {\tilde{\mu }_{v}^{(i - 1)} - \tilde{g}^{(i)} \left( {\tilde{\mu }_{x}^{(i)} ,\tilde{\mu }_{v}^{(i)} } \right)} \right) \\ \Upomega_{x}^{(i)} & = {\text{diag}}\left( {\pi_{x}^{(i)} \left( {\mu_{x}^{(i)} ,\mu_{v}^{(i)} } \right)} \right) \\ \Upomega_{v}^{(i)} & = {\text{diag}}\left( {\pi_{v}^{(i)} \left( {\mu_{x}^{(i)} ,\mu_{v}^{(i)} } \right)} \right) \\ \end{aligned} $$

Equation (3) can be derived fairly easily by computing the free energy for the hierarchical model in Eq. (2) and inserting its gradients into Eq. (1). This produces a relatively simple update scheme, in which conditional expectations are driven by a mixture of prediction errors, where prediction errors are defined by the equations of the generative model.

It is difficult to overstate the generality of Eq. (3): its solutions grandfather nearly every known statistical estimation scheme, under parametric assumptions about additive or multiplicative noise (Friston 2008). These range from ordinary least squares to advanced variational deconvolution schemes. The scheme is called *generalised Bayesian filtering* or predictive coding (Friston et al. 2010a, b): see also (Rao and Ballard 1999). In neural network terms, Eq. (3) says that error units receive predictions from the same level and the level above. Conversely, conditional expectations (encoded by the activity of state units) are driven by prediction errors from the same level and the level below. These constitute bottom-up and lateral messages that drive conditional expectations towards a better prediction to reduce the prediction error in the level below. This is the essence of recurrent message passing between hierarchical levels to optimise free energy or suppress prediction error: see (Friston and Kiebel 2009a, b; Feldman and Friston 2010) for a more detailed discussion. In neurobiological implementations of this scheme, the sources of bottom-up prediction errors are thought to be superficial pyramidal cells that send forward connections to higher cortical areas. Conversely, predictions are conveyed from deep pyramidal cells, by backward connections, to target (polysynaptically) the superficial pyramidal cells encoding prediction error (Mumford 1992; Friston and Kiebel 2009a, b).

In the present context, the key thing about this predictive coding scheme is that the precisions at each level in the hierarchy depend on the expected hidden causes and states in the level above. It is this dependency we have proposed mediates attention or selection in hierarchical inference (Feldman and Friston 2010; Friston et al. 2012). Equation (3) tells us that the state-dependent precisions modulate the responses of the error units to their presynaptic inputs. This modulation depends on the conditional expectations about the states and suggests something intuitive—attention is mediated by activity-dependent modulation of the synaptic gain of principal cells that convey sensory information (prediction error) from one cortical level to the next. This translates into a top-down control of synaptic gain in principal (superficial pyramidal) cells elaborating prediction errors and fits comfortably with the modulatory effects of top-down connections in cortical hierarchies that have been associated with attention and action selection.

### Action

In active inference, conditional expectations elicit behaviour by sending top-down predictions down the hierarchy that are unpacked into proprioceptive predictions at the level of the cranial nerve nuclei and spinal cord. These engage classical reflex arcs to suppress proprioceptive prediction errors and produce the predicted motor trajectory

4 $$ \dot{a} = - \frac{\partial }{\partial a}F = - \frac{{\partial \tilde{s}}}{\partial a} \cdot \xi_{v}^{(1)} $$

The reduction in action to classical reflexes follows because the only way that action can minimise free energy is to change sensory (proprioceptive) prediction errors by changing sensory signals; cf., the equilibrium point formulation of motor control (Feldman and Levin 1995). In short, active inference can be regarded as equipping a generalised predictive coding scheme with classical reflex arcs: see (Friston et al. 2009, 2010) for details. The actual movements produced clearly depend upon top-down predictions that can have a rich and complex structure, as we will see next.

## Simulations of sensory attenuation

This section provides a series of simulations—using the active inference scheme of the previous section—to illustrate the basic phenomena we are trying to explain. In what follows, we describe a minimal model of sensations that can be generated internally or externally. This model is used to illustrate the permissive and necessary role of sensory attenuation in the production of self-made acts. We then address the perceptual consequences of sensory attenuation, in terms of detecting externally and internally generated events—that has been the focus of much work in psychology and psychophysics reviewed in the introduction. Using the same model, we then reproduce the force-matching illusion by yoking externally applied forces to the perceived level of self-generated forces. Finally, we demonstrate the disappearance of the illusion and the emergence of false inferences about (antagonistic) external forces when sensory attenuation (attenuation of sensory precision) is removed.

### The generative process and model

**Fig. 1:**
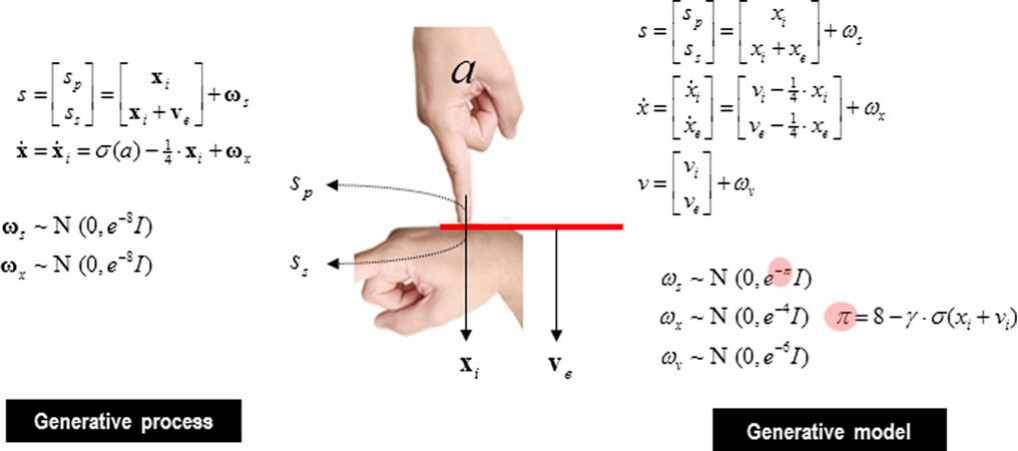
Generative model: This figure shows the generative process and model used in these simulations. The generative process (*left*) models real-world states and causes, while the generative model (*right*) is used by the subject to make inferences about causes of its sensations. In the real world, the hidden state *x*_*i*_ models self-generated forces that are sensed by both somatosensory *s*_s_ and proprioceptive *s*_p_ input channels. External forces are modelled with the hidden cause *ν*_e_ and are sensed only by the somatosensory input channel. Action causes the self-generated force to increase and is modified by a sigmoid squashing function *σ* (a hyperbolic tangent function). The hidden state decays slowly over four time bins. In the generative model, causes of sensory data are divided into internal causes *ν*_i_ and external causes *ν*_e_. The hidden cause excites dynamics in hidden states *x*_i_ and *x*_e_ which decay slowly over time as above. Internal force is perceived by both proprioceptive and somatosensory receptors, while external force is perceived only by somatosensory receptors. Crucially, the precision of the sensory prediction error *π* is influenced by the level of internal force, again modulated by a squashing function, and controlled by a parameter *γ* which governs the level of attenuation of precision. The *pink circles* highlight this state-dependent precision, which effectively controls the influence of sensory prediction errors during active inference

Figure 1 describes the generative process and model in terms of equations (that have the same hierarchical form as Eq. 2) and a schematic showing how the hidden states and causes are interpreted. This model is as simple as we could make it, while retaining the key ingredients that are required to demonstrate inference about or attribution of agency. The equations on the left describe the real world (whose states and causes are in boldface), while the equations on the right constitute the subject’s generative model. In the real world, there is one hidden state ${\mathbf{x}}_{i}$ modelling self-generated force or pressure that is registered by both proprioceptive $s_{p}$ and somatosensory $s_{s}$ input. This hidden force increases with action and decays with a time constant of four time bins (where each time bin corresponds to about 100 ms). Externally generated forces are modelled with ${\mathbf{v}}_{e}$ and add to the internally generated forces to provide somatosensory input.

The key thing about this model is that somatosensory sensations are caused ambiguously, by either internally or externally generated forces. The only way that the underlying cause of the sensations can be resolved is by reference to proprioceptive input—that is only generated internally. This is a very simple model, where the somatosensory input is being used metaphorically to stand in for the sensory consequences of events that could either be caused by self or others, while proprioceptive input represents those sensory signals that can only be caused by self-made acts. Active inference now compels the subject to infer the causes of its sensations:

The generative model used for this inference is shown on the right. In this model, internally and externally generated forces $(x_{i} ,x_{e} )$ are modelled symmetrically, where changes in both are attributed to internal and external hidden causes $(v_{i} ,v_{e} )$, with the same restorative dynamics associated with action above. The hidden causes trigger the dynamics associated with the hidden states, much like a push which sets a swing in motion. This means that proprioceptive and somatosensory inputs are explained in terms of hidden causes, where proprioceptive sensations are caused by internally generated forces and somatosensory consequences report a mixture of internal and external forces. Crucially, the precision of the sensory prediction errors depends upon the magnitude of the internally generated force (and its hidden cause). This dependency is controlled by a parameter $\gamma$ that mediates the attenuation of sensory precision: as internally generated forces rise, sensory precision falls, thereby attenuating the amplitude of (precision weighted) sensory prediction errors. These context or state-dependent changes in precision enable the agent to attend to sensory input, or not—depending upon the relative precision of prediction errors at the sensory and higher levels. This context sensitive sensory precision is shown in Fig. 1 as $\pi$.

Notice that, from the point of view of the subject, there is no real difference between hidden causes of internal and external forces—other than that the internal forces affect both proprioceptive and somatosensory inputs, while external forces only produce somatosensory sensations. Although action can fulfil proprioceptive predictions, the subject does not need to know this. In other words, it is not aware of its reflexes; it simply attributes particular sensations to particular hidden causes, which we interpret as self-generated.

### Precision and the psychophysics of sensory attenuation

In the simulations which follow, we try to reconcile the literature on stimulus detection and ratings of intensity by associating the reported intensity of a stimulus with its 90 % lower posterior confidence bound. This means that the detectability and subjective intensity are functions of both the conditional expectation and confidence or precision—such that stimulus intensity is reported to be greater when the confidence that it exceeds some threshold is larger. This is an important assumption because it implicates the subject’s confidence in the estimation of intensity and therefore speaks to a role for precision in subjective reports of sensory attenuation. Invoking a (signal detection or decision theoretic) notion of a threshold rests on the fact that sensory attenuation is only observed for stimulus attributes that can be above a threshold; for example, loudness, pressure, unpleasantness and so on. Stimulus attributes that do not have an intensity threshold could not be treated in this fashion and—we would suggest—could not show sensory attenuation. For example, although one can attenuate the loudness of an auditory tone, one cannot attenuate its frequency (which can only change by going up or down). Put simply, sensory attenuation can only be expressed in sensory modalities that have the attribute of intensity.

The relationship between physical stimulus intensity and perceived stimulus intensity is not linear. In many domains, the relationship is approximated by a power law: that is, perceived intensity is proportional to physical intensity raised to the power of an exponent (Stevens 1967). In the case of somatosensory pressure, this exponent is less than one (Xiong et al. 2013), meaning that—at higher levels of pressure—the same increase in physical pressure produces a smaller increase in perceived pressure. A clue as to why this might be is found in Weber’s law (Weber 1846), which states that the just-noticeable difference between figure luminance and background luminance increases as background luminance increases. Higher background light levels increase the amplitude of random fluctuations in the stimulus, making discrimination more difficult. It could be that this ‘diminishing returns’ effect seen in pressure perception results from higher levels of noise attenuating the perception of the stimulus.

As noted above, attentional processing can also be cast in terms of state-dependent precision. In Feldman and Friston (2010), we suggest that attention is the process of optimising precision in neural hierarchies, such that attended locations or objects are afforded high precision. This process is exactly opposite to the process of sensory attenuation described above: during sensory attenuation, attention is withdrawn from the consequences of movement, so that movement can occur. Directing attention to a stimulus can increase its perceived intensity: in the visual domain, this has been demonstrated in the cases of contrast (Liu et al. 2009; Carrasco et al. 2004; Treue 2004), colour saturation (Fuller and Carrasco 2006), speed (Turatto et al. 2007), flicker rate (Montagna and Carrasco 2006) and spatial frequency (Gobell and Carrasco 2005; Abrams et al. 2010). Given that judgements of stimulus intensity are necessarily subjective, the corollary—that withdrawing attention should decrease intensity—is entirely sensible. There is little empirical work directly addressing the effect of stimulus uncertainty (sensory precision) on perceived intensity. However, it has been demonstrated that in the auditory domain, loudness is attenuated by the addition of a noise mask (Richards 1968; Lochner and Burger 1961; Stevens 1966, 1967). We hope to address this question in the visual and somatosensory domain, in future psychophysical experiments.

### Functional anatomy

**Fig. 2:**
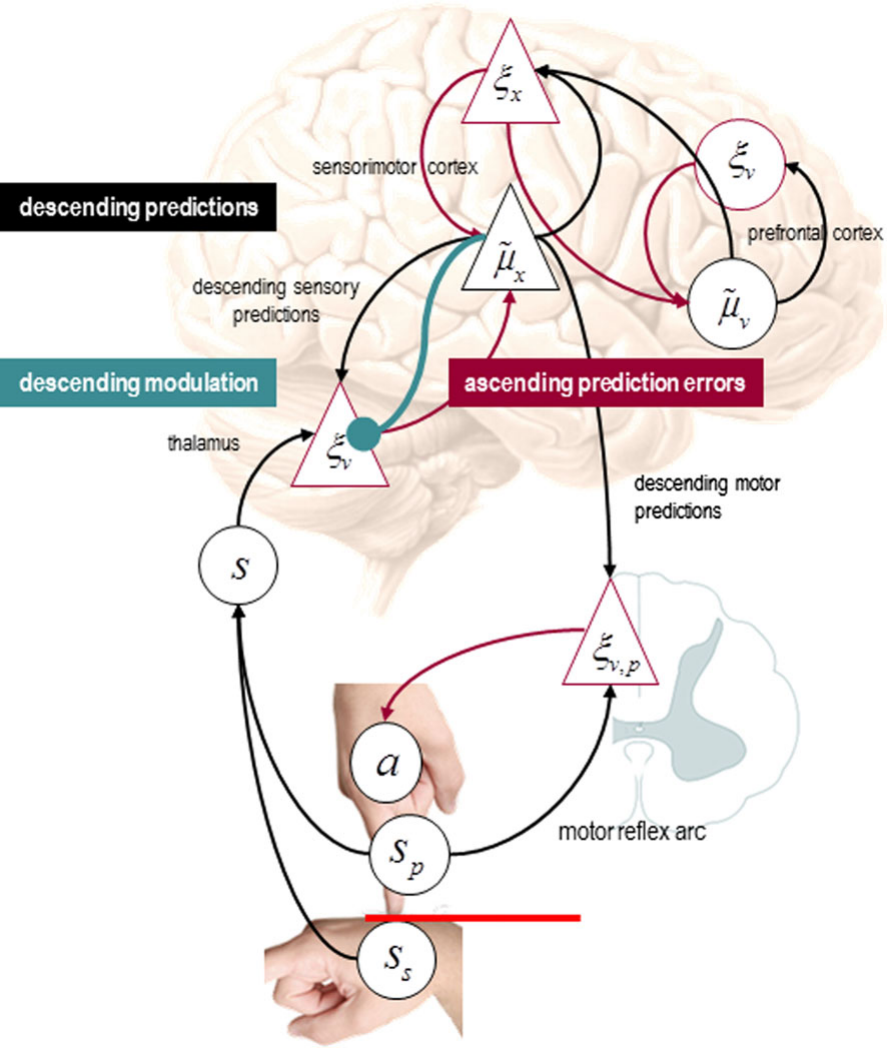
Functional anatomy: Speculative mapping of Eq. (3) onto neuroanatomy. Somatosensory and proprioceptive prediction errors are generated by the thalamus, while conditional expectations and prediction errors about hidden states (*circles*) (the forces) are placed in sensorimotor cortex. The expectations and prediction errors about the hidden causes of forces (*triangles*) have been placed in the prefrontal cortex. In active inference, proprioceptive predictions descend to the spinal cord and elicit output from alpha motor neurons (playing the role of proprioceptive prediction error units) via a classical reflex arc. Red connections originate from prediction error units (ξ cells) and can be regarded as intrinsic connections or ascending (forward) extrinsic connections from superficial principal cells. Conversely, the *black connections* represent intrinsic connections and descending (backward) efferents from (deep) principal cells encoding conditional expectations ($\tilde{\mu }$ cells). The *cyan connections* denote descending neuromodulatory effects that mediate sensory attenuation. The crucial point to take from this schematic is that conditional expectations of sensory states (encoded in the pyramidal cell $\tilde{\mu }_{x}$) can either be fulfilled by descending proprioceptive predictions (that recruit classical reflex arcs), or they can be corrected by ascending sensory prediction errors. In order for descending motor efferents to prevail, the precision of the sensory prediction errors must be attenuated

If we place this model in the predictive coding scheme above, one obtains a simple architecture that is shown schematically in Fig. 2. The precise anatomy illustrated in the figure should not be taken too seriously but illustrates how a generative model can be transcribed into a plausible neuronal architecture for predictive coding and active inference. In this particular example, we have assigned sensory prediction errors to the thalamus, while corresponding expectations and prediction errors about hidden states (forces) are associated with the sensorimotor cortex. The expectations and prediction errors about the hidden causes of forces have been placed—somewhat agnostically—in the prefrontal cortex. Notice how proprioceptive predictions descend to the spinal cord to elicit output from alpha motor neurons (playing the role of proprioceptive prediction error units) to elicit movements through a classical reflex arc. Red connections originate from prediction error units and can be regarded as intrinsic connections or ascending (forward) extrinsic connections from superficial principal cells. Conversely, the black connections represent intrinsic connections and descending (backward) efferents from (deep) principal cells mediating conditional predictions. The cyan connections denote descending neuromodulatory effects that mediate attenuation of sensory precision. The ensuing hierarchy conforms to the functional form of the predictive coding scheme in Eq. (3). In this architecture, predictions based on expected states of the world $\tilde{\mu }_{v}$ can either be fulfilled by reflex arcs, or they can be corrected by ascending sensory prediction errors. Which of these alternatives occur depends on the relative precisions along each pathway—that are set by the descending modulatory connection to sensory prediction errors. We now use this model to demonstrate some key points:

### The permissive role of sensory attenuation in action

**Fig. 3:**
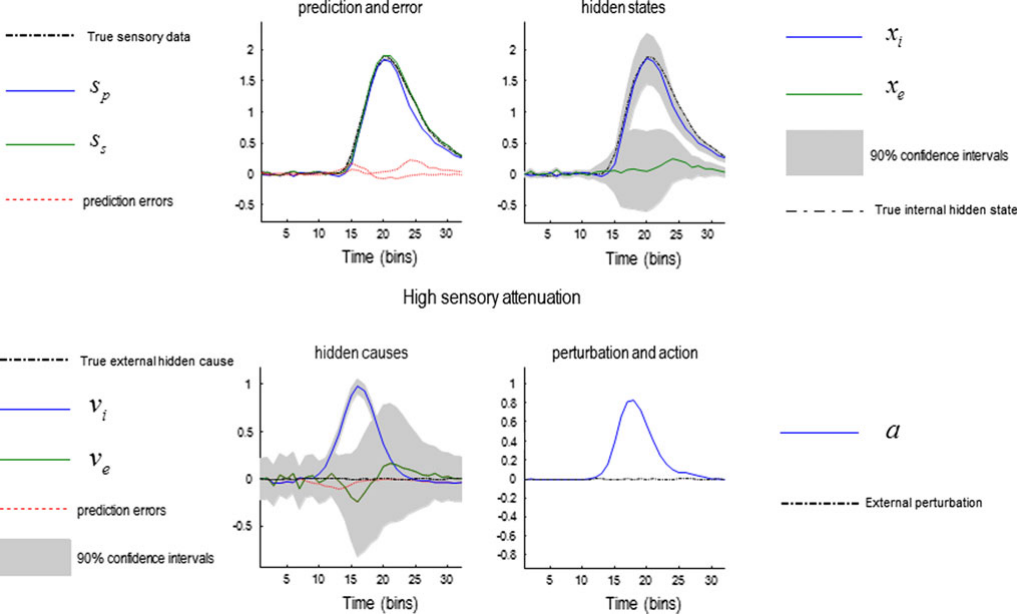
Sensory attenuation and action: simulation results illustrating the permissive effect of sensory attenuation in movement. The model was supplied with a prior belief about the hidden cause of internally generated movement, while sensory attention was high ($\gamma = 6$). This prior expectation was a simple Gaussian function of time (*blue line* in the *lower left panel*) and engenders beliefs about forces (*upper right panel*), which produce proprioceptive predictions (*upper left panel*). Action is enslaved to fulfil these predictions (*lower right panel*). Note the confidence interval around the external cause temporarily inflates during action (*lower left panel*), reflecting the attenuation of sensory precision

In the first simulations, we illustrate the necessary role of state-dependent changes in sensory precision (sensory attention) in permitting self-generated behaviour. To produce internally generated movements, we simply supplied the subject with prior beliefs that the internal hidden cause increased transiently to a value of one, with high sensory attenuation $\gamma = 6$. Figure 3 shows the results of this simulation. The lower left panel shows the internal hidden cause (blue line) with relatively tight 90 % confidence intervals (grey areas), reflecting the relatively high log precision on this hidden cause of six. Log precisions are a convenient way of quantifying confidence or certainty about prediction errors and correspond to the logarithm of the associated precision or confidence. Prior beliefs about this hidden cause excite posterior beliefs about internally generated forces, while at the same time attenuating the precision of sensory prediction errors. This is reflected by the rise in the conditional expectation of the internal force (blue line in the upper right panel) and the transient increase in the confidence interval about this expectation, due to the attenuation of sensory precision. The resulting proprioceptive predictions are fulfilled by action, and they are sensed very accurately (shown in the upper left panel). Note that proprioceptive prediction (blue line) corresponds to somatosensory prediction (green line) and that both are close to the real values (broken black line). This simulation shows normal self-generated movement under permissive sensory attenuation.

**Fig. 4:**
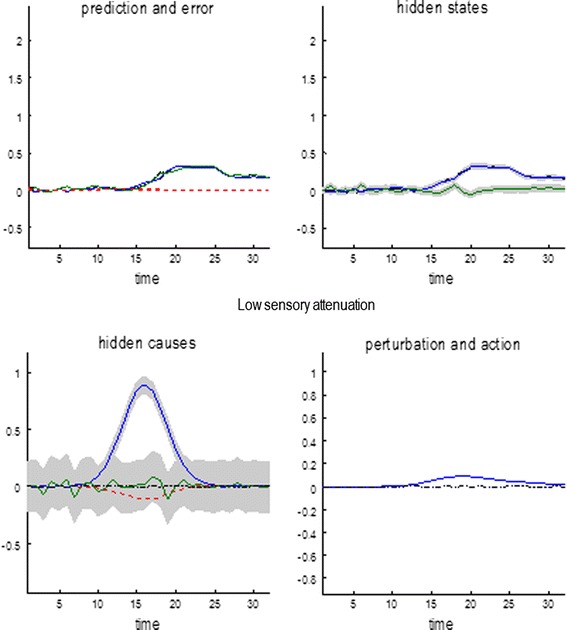
A simulation of akinesia: This figure uses the same format as previous figure but reports the results of simulations when sensory attenuation is much lower (*γ* *=* 2). In this case, bottom-up prediction errors retain a higher precision than descending predictions during movement. Conditional expectations that are updated by ascending prediction errors (*upper right panel*) overwhelm prediction errors based upon top-down predictions, and consequently infer that there is no change in the state of the world. This means that proprioceptive prediction errors are not produced (*upper left panel*) and action is profoundly suppressed (*lower right panel*)

Compare these results with the equivalent simulation when sensory attenuation was reduced from six to two (Fig. 4). Here, the sensory attenuation leaves the sensory precision higher than the precision of the prior beliefs about internal hidden causes. This means that bottom-up sensory prediction errors predominate over top-down projections, and the expected internal hidden force is profoundly suppressed—and inferred with a high degree of confidence. Because there are no predictions about proprioceptive changes, there is a consequent hypokinesia and failure of movement.

There is an interesting link between this simulation and a body of clinical, behavioural and experimental evidence regarding the impairment of movement by self-focussed attention; that is, attending the actual process of moving. Attention towards movement has been recognised as a major factor in the phenomenon of ‘choking’ under pressure in professional sportspeople, where they are sometimes rendered unable to produce over-learned movements in a performance situation (Beilock and Carr 2001). Less extreme versions of this phenomenon are part of normal experience: most of us can probably recall an incident when our movement has been impaired when we focus on it too much. This phenomenon has been described as ‘re-investment’ in movement and has been shown to impair performance and motor learning in a number of behavioural simulations (Maxwell et al. 2006; Chell et al. 2003; Zhu et al. 2011; Malhotra et al. 2012). Experimentally, asking healthy subjects to attend the production of an over-learned sequence of key presses impairs performance and elicits activation in prefrontal and anterior cingulate cortex, which is not activated during natural (unattended) sequence production (Jueptner et al. 1997). The suggestion, in the light of our model, is that attending to the sensory consequences of movement increases the precision of sensory evidence, so that descending predictions of the intended proprioceptive state are foreshadowed by precise sensory prediction error—and movement is precluded. In other words, movement is imperceptible, for both the subject and any observer.

**Fig. 5:**
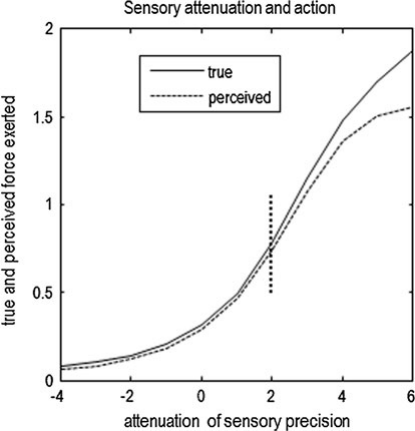
Movement and precision: True internally generated force *x*_i_ and perceived internally generated force (lower 90 % confidence interval of *x*_i_) simulated over a range of sensory attenuations, where $\gamma = \{ 6, \ldots - 4\}$. Confident movement gradually emerges as the prior precision increases in relation to sensory precision, with movement being around half its maximum amplitude when prior and sensory precisions are balanced (*γ* *=* 2, *vertical line*). Force on the *y* axis is measured in arbitrary units

Figure 5 (solid line) shows the results of simulations repeated over a range of sensory attenuations, where $\gamma$ was decreased from 6 to -4, and we recorded the internally generated force. As the prior precision increases in relation to sensory precision, prior beliefs are gradually able to incite more confident movement, with movement being around half its maximum amplitude when prior and sensory precision are in balance ($\gamma = 2$, vertical line). In short, this simple demonstration shows that sensory attenuation is necessary if prior beliefs are to supervene over sensory evidence, during self-generated behaviour. However, there is a price to be paid for the sensory attenuation, which we consider next.

### Sensory attenuation and perception

**Fig. 6:**
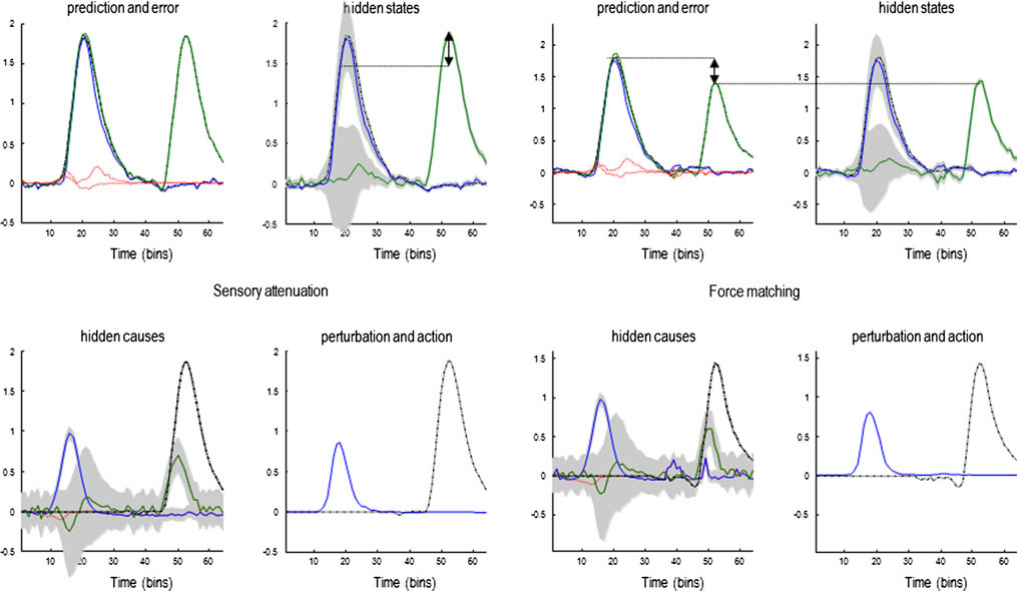
Simulation of the force-matching task. In the first part of this simulation (*left hand panels*), an internal force is generated (from a prior belief about the hidden cause *ν*_i_), followed by the presentation of an external force. The estimates of the hidden states (*upper right panel*) are similar, but the confidence interval around the force for the internally generated state is much broader. If perceptual inference is associated with the lower 90 % confidence bound of the estimate of the hidden state, it will be lower when the force is self-generated (*double-headed arrow*, *upper right panel*). This is demonstrated in the *right-hand panels*. This is a simulation the force-matching paradigm where the external force is matched to the lower bound of the 90 % confidence interval of the internal force. This means that internally generated force is now greater than the externally applied force (*double-headed arrow*, *upper left panel*)

Clearly, reducing the precision of sensory prediction errors reduces the posterior confidence in beliefs about their causes. Figure 3 shows an inflation of the posterior uncertainty (90 % grey confidence intervals) due to sensory attenuation. The consequence of this transient uncertainty—due to a temporary suspension of attention to sensory input—provides a simple explanation for sensory attenuation in terms of psychophysical detection. This can be demonstrated fairly easily by presenting the forces generated by the subject exogenously and comparing the posterior beliefs about internal and external hidden states (forces). The left panels of Fig. 6 show the results of this simulation, in which there has been a veridical inference about the succession of internal and external hidden causes (blue and green lines in the lower left panels), with a reasonable degree of confidence. Furthermore, the predictions about internally and externally generated sensations are accurate and subtended by veridical conditional expectations. However, the confidence interval around the estimate of the internal hidden state is much greater than for the external hidden state. This means that if we asked the subject to report somatosensory sensations at 90 % confidence, the externally generated sensations would be detected much more readily than the internally generated sensations. This is the essence of sensory attenuation in psychophysical studies and—in this simulation—rests upon the inflation of the confidence interval associated with internally generated consequences. In other words, we would expect a reduction in *d*-prime for events that were self-generated, relative to exactly the same events that were generated externally—as demonstrated experimentally (Cardoso-Leite et al. 2010). As this reduction in precision is applied to the entire sensory channel for the duration of the movement, a reduction in *d*-prime will also been seen for external stimuli produced during voluntary movement. This result has also been demonstrated experimentally (Juravle and Spence 2011; Van Hulle et al. 2013). This attenuation is shown by the double-headed arrow in Fig. 6. Exactly the same interpretation can be applied to the force-matching paradigm:

### Sensory attenuation and the force-matching illusion

The right-hand panels of Fig. 6 show exactly the same results as in the left hand panels; however, here, we have yoked the exogenous force to the self-generated force perceived at 90 % confidence, (as opposed to the true force exerted by the subject). In other words, the external force corresponds to the force that would be reported by the subject to match the perceived force at 90 % confidence. Crucially, the internally generated force is now much greater than the matched external force. This is the key finding in the force-matching illusion and is entirely consistent with the sensory attenuation literature mentioned above. In this setting, the loss of confidence in posterior estimates of hidden states that are self-generated translates into an illusory decrease in the intensity of percept, and hence, an increase in the force applied, relative to the equivalent force in the absence of sensory attenuation.

**Fig. 7:**
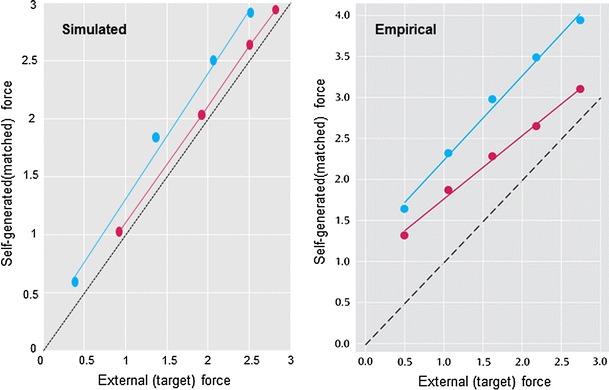
Sensory attenuation in schizophrenia: *Left panel* results of the force-matching simulation repeated under different levels of self-generated force. For normal levels of sensory attenuation (*blue circles*), internally produced force is higher than externally generated force at all levels of force, consistent with published data. Force-matching typical of schizophrenia (*red circles*) was simulated by reducing sensory attenuation and increasing the precision of prediction errors at higher levels of the hierarchy. This resulted in a more veridical perception of internally generated force (*small circles*). *Right panel* empirical results using the same format adapted (with permission) from (Shergill et al. 2003, 2005)

To simulate the force-matching paradigm, we repeated these simulations under different levels of self-generated forces by modulating the prior beliefs about the internal hidden cause (from a half to twice the normal amplitude). The results are shown in Fig. 7 (blue line) by plotting the self-generated force against the yoked or matched external force with a corresponding 90 % confidence interval. These results are remarkably similar to those obtained empirically (Shergill et al. 2003, 2005) and reveal sensory attenuation through an illusory increase in the self-generated force, relative to matched forces over a wide range of forces. In the final simulations, we ask what would happen if subjects compensated for a failure in sensory attenuation by increasing the precision of their prior beliefs.

### False inference and precision

To simulate pathology of sensory attenuation, we reduced sensory attenuation and—to compensate—increased the precision of prediction errors at higher levels in the hierarchy (by reducing sensory attenuation and increasing the log precision of prediction errors on hidden states and causes by four log units). In the absence of sensory attenuation, movement can only be elicited when there is a compensatory increase in the precision of proprioceptive predictions. In other words, beliefs about an intended movement have to be held with undue conviction (precision) to render them immune from contradictory sensory evidence that has not been attenuated.

These changes to precision mean that sensory attenuation is abolished, as indicated by the red line in Fig. 7 (left panel). This reports the results of repeating the above force-matching simulations over a range of internally generated forces but with a compensated loss in sensory attenuation. The resulting behaviour is very reminiscent of empirical results found in schizophrenia (right panel—Shergill et al. 2003, 2005). One might ask why optimal subjects do not simply adopt this strategy and use very precise prior beliefs about hidden causes?

**Fig. 8:**
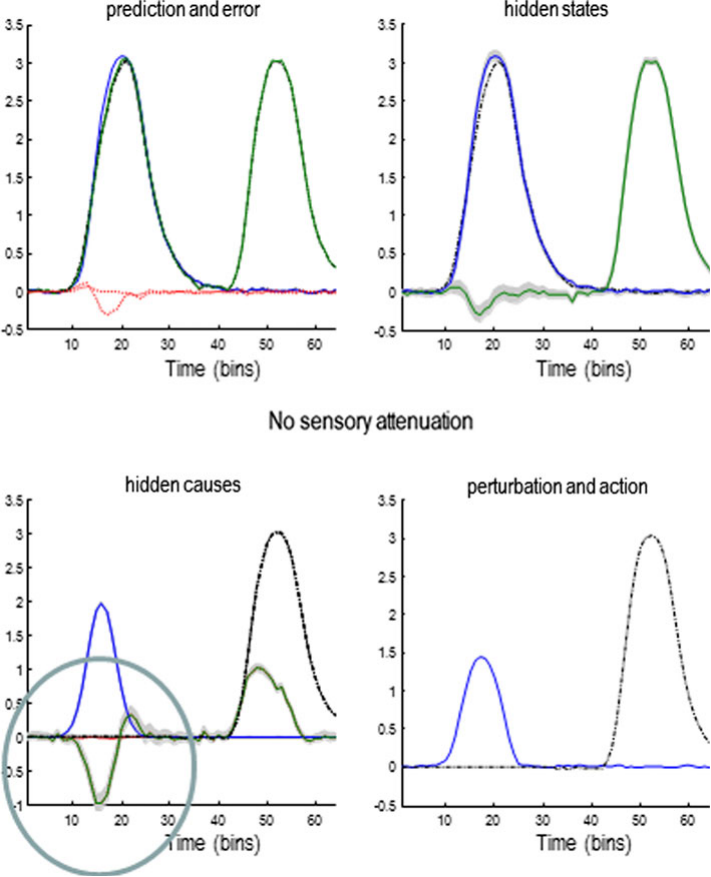
Pathology of sensory attenuation. To simulate the force-matching results seen in schizophrenia, sensory attenuation was reduced and precision at non-sensory levels of the hierarchy increased to allow movement. This results in a precise and accurate perception of internally and externally generated sensations (*upper left panel*). However, the causes of sensory data are not accurately inferred: an illusory cause (circled response in the *lower left panel*) is perceived during internally generated movement that is antagonistic to the movement. This is because the proprioceptive prediction errors driving action are rendered overly precise, meaning higher levels of the hierarchy must be harnessed to explain them, resulting in a ‘delusion’

The answer is evident in Fig. 8, which shows the results of a simulation with low sensory attenuation and augmented precisions at non-sensory levels of the generative model. Here, there is an almost perfect and precise inference about internally and externally generated sensations. However, there is a failure of inference about their hidden causes. This can be seen on the lower left, where the subject has falsely inferred an antagonistic external hidden cause that mirrors the internal hidden causes: that is, it believes that when it presses its finger on its hand, something also pushes its hand against its finger. Note that this false inference does not occur during normal sensory attenuation (see previous figures), where the true external hidden cause always lies within the 90 % confidence intervals. The reason for this false inference or delusion is relatively simple: action is driven by proprioceptive prediction errors that always report less force than that predicted (if they did not, the reflex would not be engaged). However, when sensory precision increases, somatosensory prediction errors become very precise and need to be explained—and can only be explained by falsely inferring an opposing exogenous force. In more general terms, to reconcile a mismatch between the predicted consequences of action and the state of the world that precedes action, external forces are falsely invoked. This only occurs when both the predictions and their consequences are deemed to be very precise. This false inference could be interpreted as a delusion in the same sense that the sensory attenuation is an illusion. Having said this, it should be noted that—from the point of view of the subject—its inferences are Bayes optimal. It is only our attribution of the inference as false that gives it an illusory or delusionary aspect. In the context of these simulations, the only difference between an illusion and a delusion is the level of the supposed failure of inference. Here, we have associated false inference at the perceptual level of hidden states with illusions and false inference at the conceptual level of hidden causes with delusions.

### Precision and psychopathology

Associating false inference at a conceptual level with delusions has some face validity in relation to empirical studies of the force-matching illusion. This illusion is attenuated in normal subjects that score highly on ratings of delusional beliefs. Furthermore, subjects with schizophrenia—who are prone to positive symptoms like delusions—are less susceptible to the force-matching illusion. In other words, there may be a trade-off between illusions at a perceptual level and delusions at a conceptual level that is mediated by a (failure of) sensory attenuation. A mechanistic contribution of the treatment in this paper is to link sensory attenuation with putative neurobiological mechanisms that involve neuromodulatory changes in the gain of principal cells reporting prediction error. One important candidate for this modulation is the dopaminergic system, a classical ascending neuromodulatory transmitter system (Howes and Kapur 2009).

Loosely speaking, our simulation results are entirely consistent with two known pathologies in schizophrenia: the loss of sensory attenuation and a hyper-dopaminergic drive to the striatum in acute psychosis. We demonstrated earlier that an uncompensated loss of sensory attenuation results in an inability to move (Fig. 4). This state is very reminiscent of the psychomotor poverty (catatonic) symptoms of schizophrenia (and other psychotic disorders) such as immobility, mutism, catalepsy and waxy flexibility. With waxy flexibility, patients may maintain a fixed posture for a long time, even though their limbs can be moved easily by an observer. Increased dopaminergic transmission in the striatum could increase the gain—that is, precision—of prior beliefs about the causes of internally generated behaviour and may reflect a compensation for the loss of sensory attenuation (as in our simulations above). A hyper-dopaminergic drive in schizophrenia could then lead to false inferences about external forces attributed to exogenous causes (such as in delusions of somatic passivity) or others in the acute psychotic state. Although it is overstretching the argument, it is tempting to equate the antagonistic aspect of falsely inferred hidden causes to the paranoid content of delusions that are typically seen in schizophrenia.

Our simulations have several important similarities with some recent simulations of schizophrenic motor symptoms (Yamashita and Tani 2012). In this work, the authors used a hierarchical predictive coding network to control a humanoid robot, and observed the effects of network lesions on both neural processing and behaviour. They showed that increasing the noise (i.e. decreasing the precision) in connections from higher to lower hierarchical areas could lead to catatonic motor symptoms, such as disorganised, stereotyped or loss of movements. We have seen exactly the same effects when reducing the precision of empirical priors in simulations of motor behaviour (Figure 13 in Friston et al. 2010a).

Finally, one might also speculate that the hypo-dopaminergic states seen in Parkinson’s disease would produce similar symptoms, for slightly different reasons; here, sensory attenuation might be intact, but hypokinesia may reflect prior beliefs about self-generated movement that are held with insufficient precision and are overwhelmed by sensory evidence that the patient is not moving.

## Discussion

The ideas presented in this paper suggest that attribution of agency—in an ambiguous situation—can be resolved by attenuating the precision of sensory evidence during movement: in other words, attending away from the sensations caused by self-made acts. When implemented in the context of active inference, this context–dependent attenuation provides a Bayes optimal explanation for sensory attenuation in terms of perceptual psychophysics. Furthermore, it explains the force-matching illusion and reproduces quantitative results. Finally, if attenuation is withdrawn, the force-matching illusion disappears and false (delusional) inferences about agency emerge. This is important, given the negative correlation between sensory attenuation and predisposition to delusional beliefs in normal subjects and the resistance to the force-matching illusion in schizophrenia. Active inference therefore links the neuromodulatory optimisation of precision to sensory attenuation and illusory phenomena during the attribution of agency in normal subjects. It also provides a functional account of deficits in syndromes characterised by false inference and impaired movement that are associated with abnormal neuromodulation.

This interplay between precision, attention, hierarchical inference and neuromodulation may also have important implications for functional movement disorders. We have previously suggested that functional motor symptoms can be thought of as a pathological attention to predictions about movement that is mediated by abnormally high levels of precision in the motor hierarchy (Edwards et al. 2012). The results of these simulations make the strong prediction that patients with functional movement disorders should resemble people with schizophrenia and show no force-matching illusion. We will pursue this in a subsequent work.

## Software note

The integration scheme and Matlab code producing the results reported in this paper can be downloaded as part of the academic freeware: http://www.fil.ion.ucl.ac.uk/spm/. The routines can be accessed via the graphical user interface in the DEM Toolbox (invoked by typing DEM).
